# Financial analysis of a locomotor exercise trial for post-stroke recovery: insights from the HIT Stroke Trial

**DOI:** 10.3389/fstro.2024.1425385

**Published:** 2024-09-23

**Authors:** Emily M. Hazen, Bria L. Bartsch, Sandra A. Billinger

**Affiliations:** ^1^Department of Neurology, University of Kansas Medical Center, Kansas City, KS, United States; ^2^Department of Physical Therapy, Rehabilitation Science, and Athletic Training, University of Kansas Medical Center, Kansas City, KS, United States; ^3^University of Kansas Alzheimer's Disease Research Center, Fairway, KS, United States; ^4^Department of Physical Medicine and Rehabilitation, University of Kansas Medical Center, Kansas City, KS, United States; ^5^Department of Cell Biology and Physiology, University of Kansas Medical Center, Kansas City, KS, United States

**Keywords:** rehabilitation, cerebrovascular accident, aerobic exercise, walking, recruitment, retention, clinical trial management

## Abstract

**Background:**

Navigating the complexities of post-stroke recovery trials requires addressing challenges in participant recruitment and retention and effective resource management to ensure trial success. The aim of this study was to examine the financial requirements associated with conducting the Moderate-Intensity Exercise vs. High-Intensity Interval Training to Recover Walking Post-Stroke (HIT Stroke Trial) at a single site encompassing a wide catchment area, recognizing the intricate challenges of participant recruitment and retention inherent in post-stroke recovery trials.

**Methods:**

To determine cost, study expense reports were gathered and divided into seven categories: recruitment, screening assessments, baseline assessments, intervention, outcome assessments, retention, and oversight. Categories were then further divided into chronological order for initial contact and prescreening, consenting, initial screening, and baseline testing. The 12-week intervention was divided into 4-week blocks: intervention block 1, post 4-week outcome testing, intervention block 2, post 8-week outcome testing, intervention block 3, and post 12-week outcome testing.

**Results:**

Total direct cost for site execution was $539,768 with cost per participant approximated as $35,984. Oversight costs accounted for 65.8% of the budget at $355,661. To achieve goals related to inclusive participant recruitment ($21,923) and retention ($28,009), our site costs totaled $49,932. Direct study-related costs included screening assessments ($5,905), baseline assessments ($15,028), intervention ($76,952), and outcome assessments ($36,288).

**Discussion:**

Clinical trials focusing on walking rehabilitation and exercise, particularly those requiring multiple assessment visits, demand rigorous oversight. This cost analysis provides important and critical insight into the expenses required to successfully execute an exercise-based walking rehabilitation trial in the United States.

## 1 Introduction

Stroke recovery trials face significant barriers, particularly in the recruitment and retention of participants. Previous publications, including our own (Morton et al., 2021) and others (Cramer et al., 2017; Blanton et al., 2006; Boden-Albala et al., 2015; Boxall et al., 2016) have highlighted numerous challenges in recruiting participants for stroke trials. These challenges include recruiting within specific timeframes for stroke chronicity and ensuring participants have access to reliable transportation for study visits. Additionally, stroke rehabilitation trials place significant demands on research teams, particularly in establishing robust recruitment infrastructures (Cramer et al., 2017). These infrastructures are most effectively supported by collaborative relationships between hospitals, physicians, and healthcare teams. By fostering these partnerships, study teams can better meet the challenges of participant recruitment, ensure consistent communication, and ultimately enhance the success of the trials (Morton et al., 2021). Beyond recruitment, study teams must also consider logistical challenges like providing transportation to improve both participant recruitment and retention. However, while these strategies are valuable, the financial costs associated with implementing them remain underreported. Detailing these costs is essential for providing estimates that can guide the planning and implementation of effective and sustainable stroke recovery trials (Anderson et al., 2000; Skolarus et al., 2014; Medford-Davis et al., 2016). Further, insight into cost demand allows for adequate funds to be devoted to resource procurement, study recruitment, outcome assessments, salaries, and other indirect study-related costs. The underestimation of required funds can hinder study execution and scientific rigor, lead to wasted resources, and negatively impact the clinical decision-making which influences patient care (Arenz et al., 2014; Bentley et al., 2019). This is particularly important for stroke rehabilitation interventions that focus on recovery, such as exercise treadmill training, where consistent participation and engagement are essential for achieving meaningful outcomes.

Exercise has gained recognition as a powerful non-pharmacological intervention due to its potential for substantial benefits on health and aging. Recognizing exercise as “one of the most promising interventions to delay physiological decline and extend the health span,” it is important to fully understand the associated costs of conducting exercise-based clinical trials (Erickson et al., 2023). A few studies have explored the cost demands for exercise trials in older adults (Donahue et al., 2021; Groessl et al., 2009, 2016), with one phase III, multisite, randomized controlled trial reporting an estimated cost per participant of $16,494. With a target sample size of 639 older adults in this trial, the total participant cost neared $10.5 million (Donahue et al., 2021). While the information from the older adult trials offer valuable insights, older adults may not experience the same challenges as people living with stroke. For example, transportation support is often required to promote inclusive and generalizable science in stroke, as many participants with moderate to severe impairment are unable to independently transport themselves. Additionally, many resources are often required for participant recruitment as multimorbidity affects up to 94% of individuals with stroke and may impede recruitment based on inclusion and exclusion criteria (Gallacher et al., 2019). Given this information, costs associated with exercise-based stroke trials may exceed those associated with older adult exercise trials. However, no study has investigated this topic.

By allocating sufficient resources to stroke recovery and exercise interventions, particularly in the context of support from initiatives like the National Institutes of Health (NIH) Stroke Network (Cramer et al., 2017) and other funding mechanisms, we enhance scientific rigor and reproducibility in clinical trials with the aim of influencing clinical practice and public health outcomes including overall mortality (Boyne et al., 2023; Joundi et al., 2021; Billinger et al., 2014, 2012). Documenting the cost demand of a walking rehabilitation exercise trial in chronic stroke will: (1) Provide increased insight into the financial resources required to successfully execute an exercise study, (2) Describe the monetary resources required to promote inclusive research in stroke, and (3) Allow future investigators to devote adequate finances to budget applications, study start-up, and study execution.

The High-Intensity Interval Training to Recover Walking Post-Stroke: HIT-Stroke Trial was a 12-week, National Institute of Health funded, multi-site exercise trial to determine the optimal training intensity for improving walking capacity in individuals 6-months to 5-years post-stroke. The detailed study protocol and main trial results have been published elsewhere (Boyne et al., 2023; Miller et al., 2021). The purpose of this cost analysis was to report the cost demands associated with the University of Kansas Medical Center site. As we've published previously, our site serves a large catchment area that spans our suburban and rural areas of Kansas (Morton et al., 2021). Kansas City is automobile-centric with poor availability of public transportation (U.S. Census Bureau, 2018-2022), which limits opportunities for research participation at an academic medical center. Our study team strives for inclusive science practices in our clinical trials. Here, we provide a detailed summary of costs associated with: (1) Resource procurement, (2) Recruitment, (3) Intervention delivery, (4) Outcome assessments, (5) Salaries, and (6) Indirect study-related costs, such as participant transportation. Further, we provide insight into budgeting to overcome common stroke recovery and rehabilitation research barriers, such as affordable transportation, parking considerations, navigating from parking to laboratory, and treatment compliance (Cramer et al., 2017).

## 2 Materials and methods

The HIT Stroke Trial occurred between April 3, 2018, and June 30, 2023. Licensed physical therapists completed intervention and assessment training and completed the required competencies for their respective roles (intervention or blinded assessor). Outcome assessments occurred across the 12-week intervention at baseline, 4, 8, and 12 weeks. Prior to randomization, participants successfully completed: (1) written informed consent, a medical history and medical record review, (2) the Patient Health Questionnaire (PHQ-9), (3) a 2-step command, (4) lower extremity Fugl-Meyer Motor Assessment, (5) lower limb spasticity assessment (Ashworth Scale), and (6) NIH Stroke Scale ataxia and neglect items.

At baseline, participants completed blinded assessments including: (1) a pre-visit form with repeated blood pressure measurements, (2) 10 meter walk tests at both comfortable and fastest possible speeds (Bohannon and Williams Andrews, 2011), (3) a 6-min walk test (ATS Committee on Proficiency Standards for Clinical Pulmonary Function Laboratories, 2002), (4) functional ambulation category, (5) the EuroQol-5 Dimension (EQ-5D), (6) Activities-specific Balance Confidence (ABC) Scale, (7) Patient Reported Outcomes Measurement Information System (PROMIS) Fatigue Scale (Cella et al., 2016), (8) and a maximal treadmill-graded exercise test (GXT; Boyne et al., 2017). These outcome assessments were repeated at 4, 8, and 12 weeks in addition to the Participant Ratings of Change survey. All questionnaires were administered via an in-person interview format during the baseline and outcome visits. During the 12-week intervention, participants completed three 45-min sessions per week consisting of a 3-min warm-up of overground walking, 10 min of overground training of either high-intensity interval training (HIT) or moderate aerobic training (MAT), 20 min of harness-assisted treadmill training (HIT or MAT), a second bout of 10-min overground training (HIT or MAT), and a 2-min cool down of overground walking. MAT performed continuous walking, while HIT performed 30-s intervals of maximum safe walking speed, interspersed with 30–60 s of passive recovery. Exercise intensity was continuously monitored using a Polar H7 chest strap connected to the Digifit iCardio application. Target intensity for MAT ranged from 40 to 60% heart rate reserve, and HIT, 60–95% heart rate reserve (Boyne et al., 2023; Miller et al., 2021). Lactate was also collected once a week to assess exercise intensity.

To estimate the financial costs of the HIT Stroke study at the University of Kansas Medical Center site, total site expenses were assigned to seven different categories: Recruitment, Screening Assessments, Baseline Assessments, Intervention, Outcome Assessments, Retention, and Oversight. Each of these categories includes costs related to study team effort, equipment and materials, and facilities and services used. Recruitment costs include factors such as staff time spent making recruitment calls and scheduling, newspaper advertisements, social media, time spent introducing the study in clinic and stroke support groups, and the increased cost factor for recruitment. The increased cost factor was defined as the financial effort required to randomize one participant and was calculated by dividing the number of participants randomized by total number of individuals phone screened for a randomization ratio (Donahue et al., 2021). The inverse of this ratio indicates the increased cost factor (Donahue et al., 2021).

Screening Assessments include costs for training physical therapist time and assistance from students. Baseline Assessments include testing physical therapist time and space usage for graded exercise testing in addition to walking tests. Intervention consists of cost of equipment including initial purchases and maintenance, physical therapist time for intervention delivery, and student workers. Outcome assessments include costs of administration of graded exercise tests, testing physical therapist time, and required equipment for each assessment. Retention costs include participant compensation for successful completion of each outcome testing visit, transportation costs to and from study visits, and medical translator fees. Oversight includes principal investigator, study coordinator, and physical therapist efforts for the intervention delivery and the assessor (physical therapist) who was blinded to group assignment across trial duration. Oversight activities include initial study start up activities such as initial training, training site study staff, Data Safety and Monitoring Board meetings, adverse event reporting, data queries, maintaining supplies, overall study coordination, and regulatory document submissions to the Institutional Review Board (IRB).

**Figure 1:**
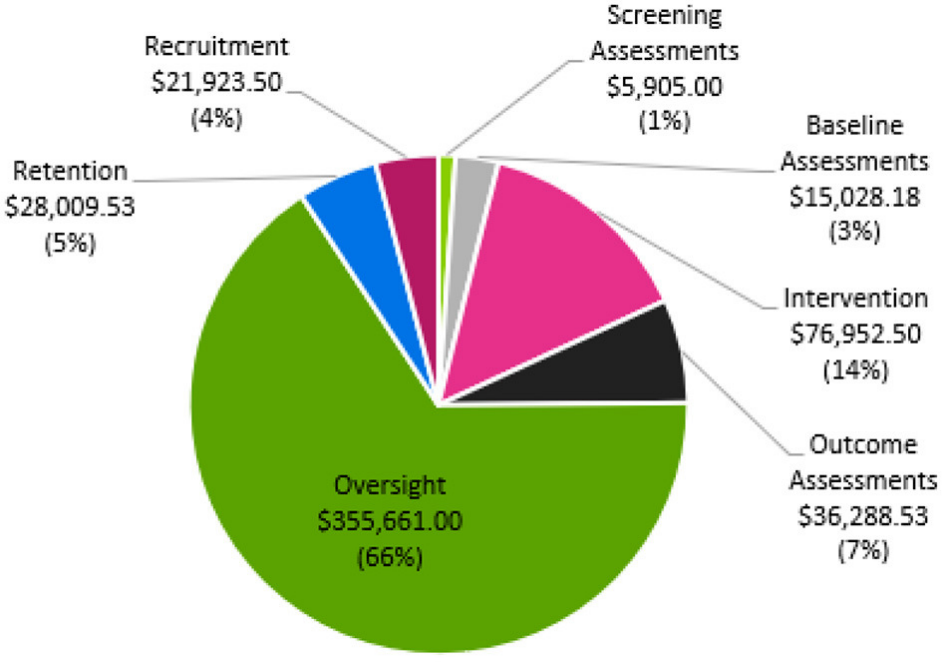
Percentages associated with trial cost distribution across categories.

To understand the distribution of these costs across the timeline of the trial, expense categories were further subdivided into chronological groups: Initial Contact and Prescreening, Consenting and Screening, Baseline Outcome Testing, Intervention Block 1 (weeks 1–4), Post-4 Week Blinded Outcome Testing, Intervention Block 2 (weeks 5–8), Post-8 Week Blinded Outcome Testing, Intervention Block 3 (weeks 9–12), and Post-12 Week Blinded Outcome Testing (Figure 1). To represent the cost per completed participant (*n* = 15), the total cost for each chronological group was divided by 15.

## 3 Results

**Figure 2:**
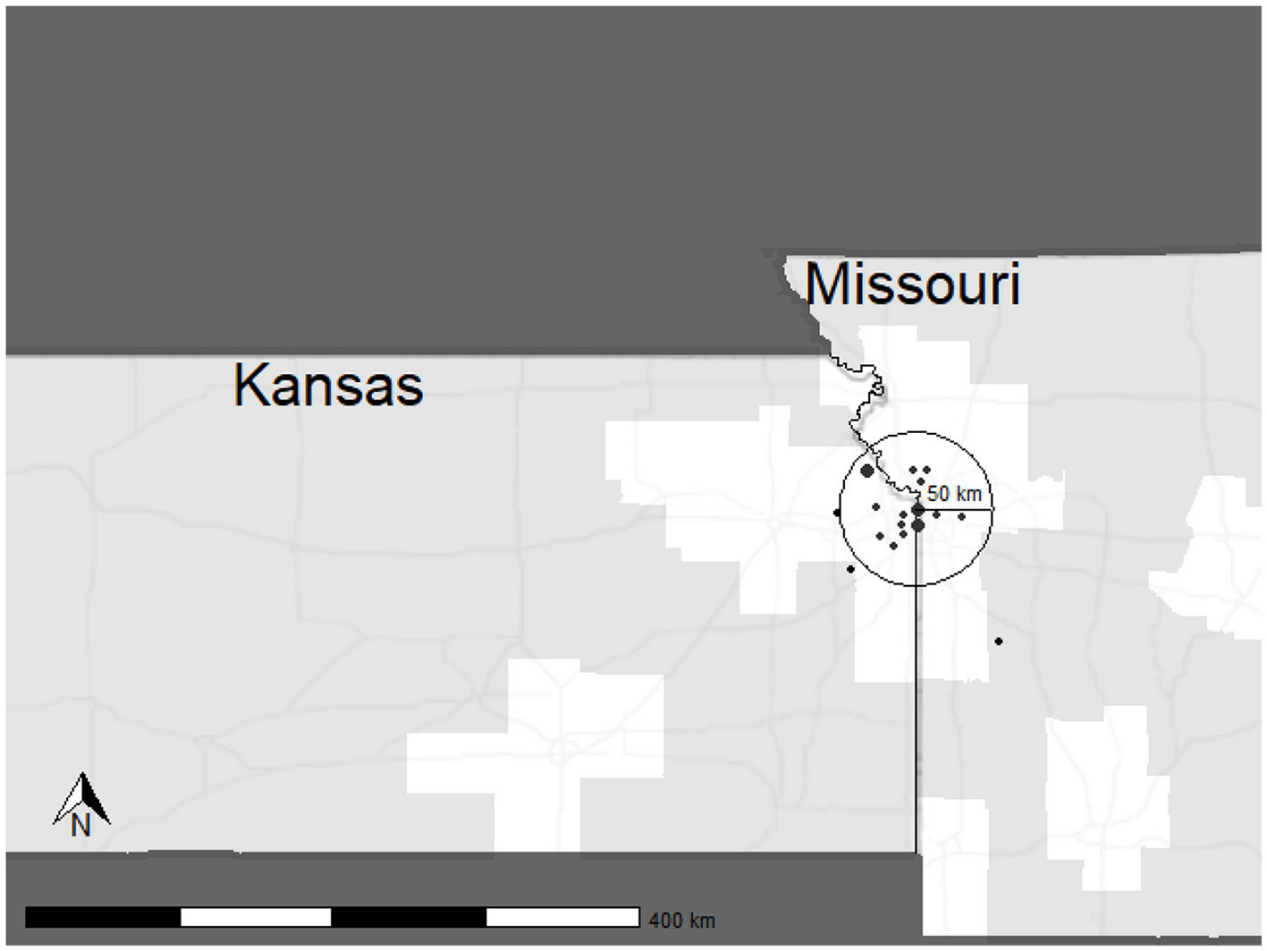
Distribution of participants enrolled in the HIT Stroke Trial. The circle depicts a 50 km radius to the laboratory with predominantly urban and suburban areas. Points are in arbitrary locations, equidistant to the laboratory to protect participant confidentiality. Point size indicates the number of participants recruited from the given area.

A total of 18 participants were enrolled at our site, with 15 completing the study. During the study, three participants withdrew due to adverse events (Boyne et al., 2023). These participants withdrew after completing Post-4 Week Blinded Outcome Testing and were included in cost analysis for all visits completed. As shown in Figure 2, most participants lived within a 50 km radius to the laboratory site. Two participants resided in rural, or frontier counties, which are indicated by light gray shading.

### 3.1 Total cost

The estimated total direct cost of the HIT Stroke Trial at the University of Kansas Medical Center was $539,768, resulting in a trial cost of $19,600 over the grant budget. This amount was offset using internal funds to support transportation costs that exceeded our site budget and to support a medical translator at each study visit and outcome testing. Figure 1 shows the percentages associated with the distribution of costs across oversight, recruitment, retention, and outcome assessment categories.

### 3.2 Cost per participant

**Figure 3:**
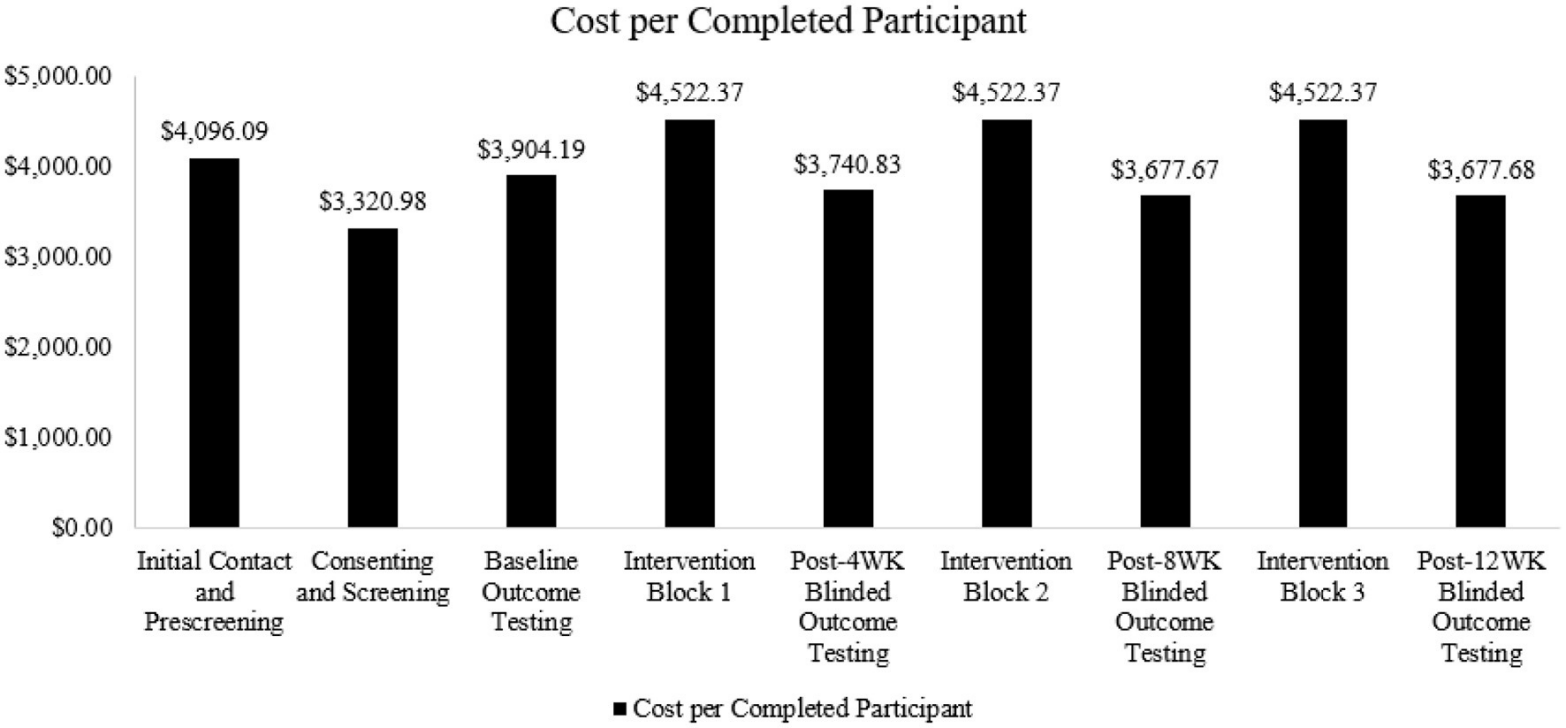
Cost for one enrolled participant to complete each trial phase.

The overall cost for one enrolled participant to complete the study was estimated to be $35,984.54. This cost includes screening, consenting, baseline and outcome testing, and intervention sessions. Oversight and retention costs have also been integrated into these categories to capture the commitment of both the principal investigator and study coordinator. Additionally, these categories encompass costs specifically allocated to enhance research accessibility. Figure 3 outlines the distribution of cost per participant.

### 3.3 Recruitment

Recruitment costs totaled $21,923, including the cost of newspaper advertisements ($530.00), flyers ($100.00), time spent introducing the trial in clinic and support groups ($19,756.00), and staff time spent conducting phone screenings ($1,537.50). As highlighted in Figure 3, cost is frontloaded per participant, and our findings indicated an increased cost factor of 5.88. This factor indicates that to randomize one participant, ~6 potential participants had to be screened.

### 3.4 Screening and baseline assessments

Initial in-person screening assessments had an estimated cost of $5,905 which included physical therapist time and student assistants in the doctor of physical therapy program. Baseline assessment costs totaled $15,028. These assessments were completed at the University of Kansas Clinical and Translational Science Unit (CTSU) with a designated physical therapist, costing ~$11,352. The cost for student assistants was ~$3,675.

### 3.5 Intervention

The cost to conduct the HIT Stroke Trial intervention at the University of Kansas Medical Center was ~$76,952. Training visits were completed by designated physical therapists, whose effort totaled at $49,756 and the student assistants resulted in a cost of ~$3,675. Equipment required for the intervention was estimated to cost approximately $23,521, including items such as a treadmill ($8,115.00), harness system ($4,817.49), heart rate monitors ($745.00), and iPods ($760.00), along with other essential equipment.

### 3.6 Outcome assessments

Outcome assessments had an estimated cost of $36,288 and were completed at our University of Kansas CTSU by assessors blinded to group assignment. Outcome assessment costs included: (1) the bundled cost for the graded exercise tests with gas analysis and personnel ($11,558.03), (2) physical therapist cost for ensuring participant safety with the treadmill and harness system throughout the graded exercise test ($21,054.75), and (3) DPT student assistance ($3,675.75).

### 3.7 Retention

**Figure 4:**
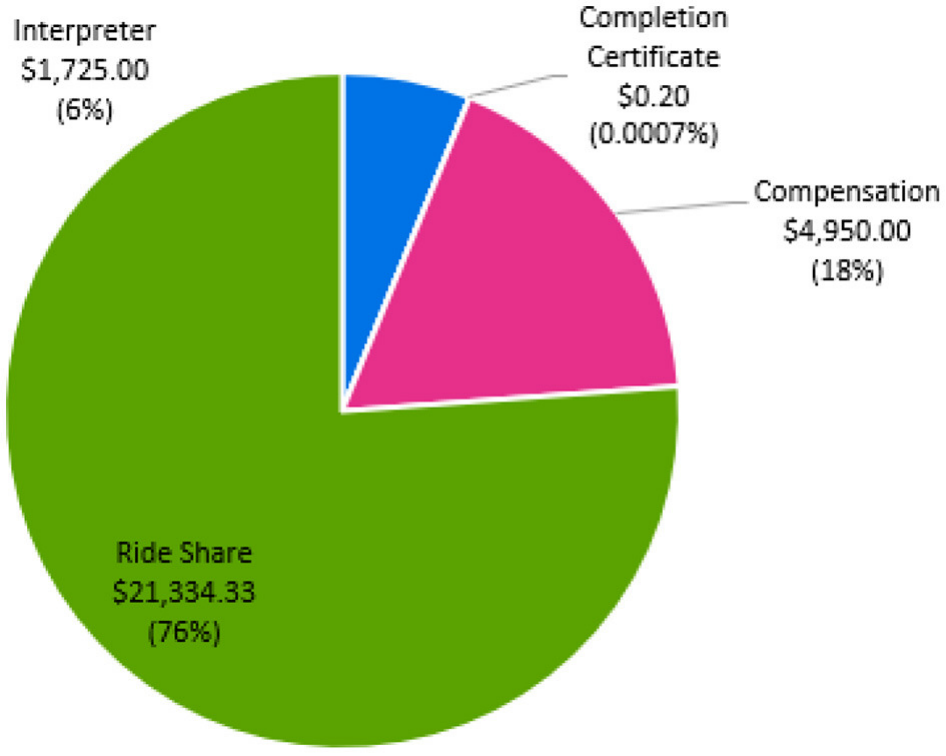
Cost of participant retention.

Figure 4 details the cost distribution related to participant retention. Retention costs totaled $28,009. Our site spent 76.1% ($21,334) on ride-share transportation to minimize the barriers of study participation. At study completion, all participants required transportation to a single study visit, highlighting the importance of providing transportation to minimize missed study visits or outcome testing. The primary reasons for transportation were: (1) family member unavailable, (2) car broke down, or (3) family member ill and unable to drive participant. Approximately, 25% of participants required transportation for all visits due to no transportation access including public transportation. We calculated ~$1,725 was spent on medical translation and interpreter services for one non-English speaking individual. For compensation, participants were provided $75 after each outcome testing visit, totaling $4,950. For study completion, participants were provided a completion certificate, for a total of $0.20.

### 3.8 Oversight

Oversight costs for the trial duration totaled $355,661 and reflects principal investigator and study coordinator total compensation. Principal investigator effort was dedicated to all trial related activities including but not limited to budget, training study team members, personnel oversight, data monitoring, study meetings, creating annual reports for our site, and data safety and monitoring board preparation and meetings, totaling $215,273. Study coordinator salary was dedicated to managing regulatory documents including institutional review board, protocol adherence, assisting with consenting, adverse event documentation, screening and outcome assessments, and participant scheduling for a total of $140,388.

## 4 Discussion

This cost analysis aimed to elucidate the financial intricacies involved in recruitment, enrollment, outcome testing, and intervention for a clinical exercise trial focusing on stroke recovery. Understanding these costs is crucial for effective resource planning, budgeting, and fund procurement as stroke rehabilitation and recovery trials “are not simply acute stroke studies that are initiated at late time points” (Cramer et al., 2017). Rather, our findings suggest that the challenges associated with recruitment, retention, and intervention delivery are unique to stroke recovery and the information provided here should address potential costs and provide valuable information related to stroke recovery trials.

### 4.1 Increased cost factor and recruitment

The data showed an average cost of enrollment and trial completion per participant was $35,984 with ~1/5 of cost dedicated to recruitment and screening for initial enrollment. This cost per participant is lower than in an acute endovascular intervention where the cost per participant in the intervention group was reported at $126,494 and $143,331 in the control (Van Den Berg et al., 2022). These costs are the result of the surgical intervention and thrombolysis associated with the trial in addition to rehabilitation therapy services, home health visits, physician consultations, and over the counter medication expenses during the 2-year follow up. As such, the difference between participant costs in our trial vs. the endovascular trial may be in part attributed to the consideration for medical follow-up and expenses following the initial endovascular intervention.

For the present study, the increased cost factor indicated our site would conduct phone screens on at least six potential participants for one to be randomized. The increased cost factor reported here is similar to that observed in a recent 12-month exercise intervention trial in older adults, which reported an increased cost factor of 5.95 for phone screening and randomization for 494 participants (Donahue et al., 2021). It is important to note, however, that factors such as study inclusion and exclusion criteria, as well as individual interest in a given study topic (Weerasekara et al., 2021), may influence the increased cost factor. For example, a study with more strict inclusion and exclusion criteria may require increased participant screening for randomization, and these factors should be considered in study design. Further, successful recruitment methods may be implemented to minimize increased cost factor. We have previously published our approach for optimizing recruitment in stroke recovery trials, using a “service first” approach (Morton et al., 2021). Establishing supportive relationships with physicians and providing training to the recruitment team on effective participant communication before study start-up may optimize recruitment (Morton et al., 2021). Further, sites may consider using participant databases for a streamlined recruitment approach. Since completion of the HIT Stroke Trial, our site has created a Stroke Recovery Registry in which we enroll participants interested in stroke recovery research. In our registry, we record demographics, medical history, and study interests to pair individuals who have experienced a stroke with potential research opportunities that meet their interests and needs based on inclusion and exclusion criteria to identify more quickly those who may be eligible during the phone screening phase.

### 4.2 Baseline and outcome assessments

Another driving factor for our site related costs in the HIT Stroke Trial was baseline and outcome testing, including the personnel time dedicated to these measures. Maximal exercise testing in research commonly utilizes an exercise physiologist with or without assistance from a research assistant. However, conducting maximal treadmill-graded exercise tests in stroke requires a multidisciplinary approach with specialized equipment to ensure participant safety and test optimization. All participants in the HIT-Stroke Trial performed testing with a bodyweight support harness and received continuous guarding and physical therapist oversight to ensure that the test was stopped if any gait patterns emerged that could lead to participant injury. The exercise physiologist conducted metabolic cart testing and determined test cessation based on physiologic criteria, such as reaching a plateau in oxygen uptake.

Given the necessity for specialized oversight, maximal exercise testing in stroke requires increased cost allotment, compared to traditional exercise testing. The annual physical therapist salary in the Kansas-Missouri area ranges from $52,690 to $93,780 (U.S. Bureau of Labor Statistics, 2023b), $55,610–$63,970 for an exercise physiologist (U.S. Bureau of Labor Statistics, 2023c), and $52,030–$59,230 for a research assistant (U.S. Bureau of Labor Statistics, 2023a). As such, requiring physical therapist oversight increases cost requirements. In addition to exercise testing, the physical therapist conducted the 10-meter and 6-min walk tests to ensure safety and provide rehabilitation expertise. Use of a physical therapist increases the ability of this research to be translated to clinical settings where physical therapy would be the primary profession implementing the HIT-Stroke Trial protocol.

While cost analyses for rehabilitation trials in stroke are nascent, a cost analysis exploring costs for a phase III, multi-site, exercise trial in older adults (Investigating Gains in Neurocognition in an Intervention Trial of Exercise; IGNITE) has been published. Compared to IGNITE, which reported 38% of total costs being allocated to outcome assessments, our trial reported only 7%. This discrepancy could be in part due to IGNITE's usage of neuroimaging (magnetic resonance imaging and positron emission tomography), which authors reported as their most expensive outcome assessments. Further, IGNITE was a 12-month trial, compared to HIT-Stroke which was 12-weeks. As such, intervention duration may affect cost allocation.

### 4.3 Intervention

For exercise sessions, a treadmill and bodyweight support harness, heart rate and lactate monitors, iPods for real time monitoring of heart rate, and step watches for session step count were required. Personnel costs included licensed physical therapists to deliver the intervention, and we employed doctor of physical therapy students to assist with the intervention. We acknowledge that our cost factor is likely influenced by the salaries associated with the need for licensed physical therapists vs. trained research assistants. However, to ensure safe delivery of the intervention and optimize the ability of this intervention to be translated to clinic, we believe these costs are justified. Physical therapists are experts in providing continuous guarding to decrease fall risk during the intervention and in identifying gait patterns which may be of concern for injury. Given the education and clinical experience required to understand the nuances of intricate gait patterns following a stroke, expecting a research assistant to acquire this knowledge without formal education would be unjustified. Further, and as previously mentioned, the use of physical therapists increases the ability of this research to translate to clinical settings.

The intervention cost for IGNITE (10% of total costs) was comparable to the intervention costs (14%) in our trial. However, the per participant cost for intervention completion was $3,401 in IGNITE, compared to $13,567 in HIT-Stroke. This cost discrepancy may be in part due to IGNITE using a small group exercise format with an exercise trainer supervising 3–4 participants per session vs. the one-on-one supervision provided by a physical therapist in HIT-Stroke. In a separate physical activity intervention trial in older adults, supervised group exercise was provided 3 times per week for 8 weeks by an exercise instructor, followed by 2 times per week for 16 weeks, and then participants were encouraged to exercise on their own with an option for group exercise 1 time per week for up to a year after beginning the trial (Groessl et al., 2009). The average cost per participant was $1,134. As such, utilizing one-on-one supervision with a rehabilitation specialist may significantly increase intervention cost requirements (Groessl et al., 2009).

Another important consideration is how geographic location may affect intervention cost, as this cost is largely influenced by interventionist salary. As cost of living varies between regions, so does physical therapist salary. In comparison with our partner sites, the annual mean wage of physical therapists is higher in Ohio and Delaware than the Kansas-Missouri region (U.S. Bureau of Labor Statistics, 2022). As such, this may be an additional consideration when planning multi-site trials.

### 4.4 Participant retention

#### 4.4.1 Transportation

Our retention costs include expenses required to decrease barriers to research engagement. Approximately $21,334 were dedicated ride share services. Research suggests that transportation is a key barrier to research participation and results from decreased access to vehicles or public transportation, inadequate infrastructure, and travel distance and costs (Rigatti et al., 2022; Health Research and Educational Trust, 2017; Cramer et al., 2017; Weerasekara et al., 2021). Our site experiences challenges for participant transportation due to the poor availability of public transportation in the Kansas City Metro area. According to Walk Score, Kansas City is a car-dependent city, with a walkability score of just 32 out of 100. Additionally, poor public transit is reflected in a score of 19 out of 100 (Walk Score, 2024). Therefore, ensuring our participants have access to transportation is critical for inclusive science. Figure 2 shows the distribution of participants for the HIT Stroke Trial in our catchment area and the willingness of people living with stroke to engage and participate in an exercise trial designed to improve walking. In advance of the clinical trial, we budgeted for participant transportation. However, our expenses related to transportation costs exceeded the budgeted amount in the grant. Internal funds and other sources of funding were used to support participants and ensure access for all. While all researchers may not have the ability to secure additional funding, transparency in costs required for transportation may improve budgeting for this resource.

In addition to transportation support, our team placed special emphasis on parking and navigating from parking locations to clinic, which have been cited as commonly neglected barriers to research engagement in stroke (Cramer et al., 2017). Our site offered free parking to all participants in the parking site nearest our laboratory, which is < 500 feet from the building entrance. To provide this parking accommodation, a staff member allotted time each week to send a list of participant first names to our parking services. We provided a detailed parking map and instructions prior to study visits. Additionally, if needed our staff would meet participants at their vehicles and provide wheelchair assistance to reduce fatigue prior to the study visit or assessment visits.

#### 4.4.2 Translation services

To further decrease barriers to engagement at our site, we used other funding sources to cover costs of $1,725 to a medical translator and language interpreter to decrease barriers to clinical trial participation. Approximately 10% of adults in the Kansas City metro area speak a language other than English at home, demonstrating a need for language services to promote inclusive recruitment and participation. The English predominance in research not only decreases research accessibility, but also the generalizability of research findings for individuals who do not speak English (U.S. Census Bureau, 2022). Research suggests that these language barriers perpetuate healthcare inequities and may negatively impact healthcare policies and delivery (Ransing et al., 2023). By providing transportation through ride-share programs and translator services, we minimize recruitment and retention barriers for all, create a trust-worthy environment within our academic medical center setting, and assist with place-based disparities for those in rural or urban settings without access to transportation.

#### 4.4.3 Compensation

Participants were compensated $300 each for their time in the HIT Stroke Trial. Although concerns have been raised regarding the potential for participant bias in studies which offer compensation (Pandya and Desai, 2013), research suggests that compensation helps to promote participant retention (Robinson et al., 2007), and offset the financial burden placed on participants for study engagement (Bierer et al., 2021). Participants often incur expenses as a result of study travel and time away from work for participation and providing study compensation can reduce socioeconomic disparities in research, where the burden of engagement is greater on those with lower income (Bierer et al., 2021).

In comparison to IGNITE which paid participants $805 for attending outcome assessments, we provided participants with four $75 reimbursements following completion of each block of outcome assessments. Providing participants with multiple reimbursement points may help to offset financial burden incurred throughout study engagement.

#### 4.4.4 Non-financial incentives for participation

Lastly, we provided participants with a certificate of study completion. Research suggests that non-financial incentives which express appreciation for participation are commonly used in studies and help promote retention (Robinson et al., 2007). Due to financial limitations in clinical trials, consideration of non-financial incentives may be optimal for helping to reduce participant drop out and loss to follow-up bias (Robinson et al., 2007).

These data provide novel findings related the cost associated with conducting a stroke recovery trial. While we tracked study related costs, we acknowledge that the cost per participant and increased cost factor are estimates, as it is impossible to determine how many individuals were reached through study advertisements or actual effort was spent on each category such as phone screens, emailing or calling participants and coordinating with the ride-share programs. Further, we report costs for a single site of the HIT Stroke Trial. Costs may differ between sites due to factors such as staff salaries based on regional cost of living, costs for space usage, transportation needs, and translation services. We did not account for costs that may have been associated with operations during the COVID-19 pandemic including personal protective equipment, additional staffing, screening participants for COVID-19, time lost due to staff or participants who reported COVID-19 exposure and institution approved cleaning solutions to protect against COVID-19. Additional costs that were not accounted for at our site include additional staff for data checking and time spent on coordinating delivery of equipment and supplies and the set-up of equipment. Finally, we acknowledge these costs are associated with trial execution in the United States. Therefore, some costs may not be relevant to all locations across the world. By emphasizing that well-funded trials yield more accurate and generalizable results, researchers can make a strong case for targeted investments that ultimately improve public health outcomes and reduce long-term healthcare costs, especially in low- and middle-income countries.

## 5 Conclusion

This analysis outlines the costs required to conduct a single blind randomized controlled trial focused on exercise and stroke recovery. We provide a detailed review of the costs associated with recruitment, screening assessments, baseline assessments, intervention, outcome assessments, retention, and oversight over the 12-week trial period. We discuss the financial requirements associated with promoting inclusive science and overcoming research barriers. The cost analysis of this trial may provide increased insight for researchers seeking to optimize stroke recovery research with inclusive science when designing budgets.

## Data Availability

The raw data supporting the conclusions of this article will be made available by the authors, without undue reservation.
